# Physical Complaints Decrease after Following a Few-Foods Diet in Children with ADHD

**DOI:** 10.3390/nu14153036

**Published:** 2022-07-24

**Authors:** Lidy Pelsser, Tim Stobernack, Klaas Frankena

**Affiliations:** 1 ADHD Research Centre, 5624 JE Eindhoven, The Netherlands; 2 Department of Animal Science, Wageningen University and Research, 6708 WD Wageningen, The Netherlands; tim.stobernack@radboudumc.nl; 3 Adaptation Physiology Group, Wageningen University and Research, 6708 WD Wageningen, The Netherlands; klaas.frankena@wur.nl

**Keywords:** ADHD, few-foods diet, children, physical complaints, atopic constitution, thermoregulation, gastrointestinal complaints, eczema, sleep problems, nutrition

## Abstract

Attention-deficit hyperactivity disorder (ADHD) symptoms may significantly decrease after following a few-foods diet (FFD). The results of a small randomised controlled trial (RCT) showed that co-occurring physical complaints in children with ADHD decreased as well. To further investigate the effect of an FFD on physical complaints, we analysed unpublished data from previously published studies (i.e., ‘Impact of Nutrition on Children with ADHD’ [INCA], an RCT, and ‘Biomarker Research in ADHD: the Impact of Nutrition’ [BRAIN], an open-label trial). In both trials, the association between an FFD, ADHD, and 21 individual physical complaints was assessed. Children either followed a 5-week FFD (the INCA FFD group and BRAIN participants) or received healthy food advice (the INCA control group). The ADHD rating scale and a physical complaint questionnaire were filled in at the start and end of the trials. The INCA results showed, for 10 of 21 complaints, a clinically relevant reduction in the FFD group compared to the control group. The open-label BRAIN results confirmed the outcomes of the FFD group. No association was detected between the decrease in physical complaints and the decrease in ADHD symptoms. The results point toward an association between the FFD and a decrease in thermoregulation problems, gastrointestinal complaints, eczema, and sleep problems.

## 1. Introduction

Attention-deficit hyperactivity disorder (ADHD), characterised by inattentive and/or hyperactive and impulsive behaviour [1], is a common mental disorder diagnosed in 6% of children [2]. ADHD is a long-lasting condition [3]; in at least 60% of children with ADHD, the impairments persist into adulthood [4]. Other mental disorders, such as oppositional defiant disorder and autism spectrum disorder, often co-occur with ADHD [5]. In addition, medical conditions such as gastrointestinal and urogenital problems are more frequently diagnosed in persons with mental disorders than in control groups [6]. A longitudinal study following children from early childhood to adolescence showed significant associations between ADHD symptoms and several physical conditions, such as asthma, food allergies, sleep problems, obesity, and infections [7]. This was confirmed in a large Danish cohort study that followed children up to 12 years of age [8].

Various genetic and environmental factors may contribute to ADHD [4]. The current consensus is that none of these factors is either a necessary or sufficient cause, although specific risk factors, such as rare genetic variants and environmental factors, can have a major impact [4]. Research has shown that nutrition can be an environmental risk factor of major impact in (a subgroup of) children with ADHD: double-blind placebo-controlled research, randomised controlled trials (RCTs) and open trials investigating the effect of a few-foods diet (FFD) on ADHD have shown that ADHD symptoms may significantly decrease after following a 5-week FFD [9,10,11,12,13,14]. These results suggest that children with ADHD who show clinically relevant behavioural improvements—i.e., a symptom decrease of at least 40% on the ADHD Rating Scale (ARS)—after following an FFD, can be diagnosed with food-induced ADHD [14].

In previous studies investigating the impact of the FFD on ADHD, the effect on often co-occurring physical complaints (amongst others: headache, asthma, rhinitis, and gastrointestinal problems) was evaluated as well, and it was reported that some of these complaints decreased after following the FFD [15,16]. Subsequently, a small RCT was conducted in which 24 children with ADHD participated: 13/24 in the intervention group following the FFD, and 11/24 in the control group adhering to their diet as usual [17]. This study was the first study to investigate, in a structured way, physical complaints in children with ADHD following an FFD. A significant decrease in the number of physical complaints was shown in the FFD group (Cohen’s d pre-post = 2.0, *p* < 0.001), while no significant difference was detected in the control group (effect size pre-post = 0.2, *p* = 0.08). Thereafter, the association between an FFD and physical complaints was investigated in two other studies, i.e., the ‘Impact of Nutrition on Children with ADHD’ (INCA) study, an RCT [14], and the ‘Biomarker Research in ADHD: the Impact of Nutrition’ (BRAIN) study, an open-label trial [18].

We analysed unpublished data from both the INCA study and the BRAIN study to verify the previous study results. The objectives were:

To assess the effect of the FFD on individual physical complaints in children with ADHD.

To evaluate the association between ADHD symptom change, atopic constitution, and change in physical complaints following the FFD.

## 2. Materials and Methods

### 2.1. Subjects and Design

In both the INCA study and the BRAIN study, children were recruited via health care institutions and the media. All included children were diagnosed with ADHD by a paediatrician specialised in ADHD. None of the children received stimulant or other ADHD treatment while participating in the studies. An extended overview of the inclusion and exclusion criteria for each study is provided elsewhere [14,18]. In short: INCA, a randomised controlled trial, included 100 children, both boys and girls, aged 4–8 years. Children were randomised either into the intervention group (*n* = 50) who followed the 5-week FFD or into the control group (*n* = 50) who received healthy food advice [14]. BRAIN, an open-label trial, included 100 boys aged 8–10 years. All children followed the 5-week FFD [13,18].

INCA and BRAIN applied the same design: after the baseline assessments (T0) to determine whether a child was eligible to participate in the study, a 2-week baseline period followed during which parents had to keep a diary and closely observe their child’s behaviour. After the baseline period, the second assessment (T1) took place. Following T1, either the control period (INCA only) or the 5-week FFD started. At the end of the FFD or control period, the third measurement took place (T2). An extensive overview of study design and FFD composition has been published elsewhere [13,14].

### 2.2. Interventions

The FFD, which was applied in the INCA FFD group and in all BRAIN participants, is a 5-week very restricted elimination diet consisting of only a few foods, i.e., rice, turkey, some vegetables, water, rice drink with added calcium, olive oil, ghee, pear, and salt. At the start of the FFD, some additional foods were allowed, such as lamb, wheat, corn, fruits, and potatoes. If no behavioural improvements were reported within 2 weeks, the diet was gradually restricted to the most stringent version of the FFD described above. Throughout the FFD, parents had to keep a comprehensive food and observation diary. 

The INCA control group received healthy food advice. Comparable to the FFD group, parents of children in the control group had to keep a comprehensive food and observation diary.

### 2.3. Questionnaires

Two questionnaires were applied, namely the physical complaint questionnaire (PCQ) [17] and the ARS [19]. All questionnaires were filled in by the researchers based on parental reports. Two timepoints, i.e., before starting the FFD (T_start_) and at the end of the FFD or control period (T_end_), were included in this study. At both timepoints, parents had to consider the complaints observed during the past week only. However, complaints that were reported at T_start_ had to also be present during the past 6 months to preclude ratings of coincidental complaints.

The INCA PCQ assessed 21 complaints, which were complemented in the BRAIN study with 2 additional complaints (i.e., daytime urinary incontinence and faecal incontinence) based on recent literature establishing an association between mental disorders such as ADHD and functional incontinence [20] (Table 1).

In the BRAIN study, all items were rated on a four-point scale based on the frequency of occurrence, i.e., ‘every day’ (=3), ‘several times a week’ (=2), ‘once a week’ (=1), and ‘less than once a week’ (=0). The PCQ in the INCA study applied a slightly different scale, i.e., ‘every day’ (=4), ‘several times a week’ (=3), ‘once a week’ (=2), ‘once a month’ (=1), and ‘never’ (=0). For comparison’s sake, we adapted the INCA PCQ rating as follows: every day (=3), several times a week (=2), once a week (=1), and less than once a week (=0). For both BRAIN and INCA, a complaint was deemed present when rated ‘several times a week’ or ‘every day’ and absent when the score was ‘once a week’ or ‘less than once a week’. All analyses were performed based on presence/absence. The complaint scores are provided in the DANS data set (https://doi.org/10.17026/dans-zgw-3yqc (accessed on 22 July 2022)).

ADHD was assessed by means of the ARS, consisting of the 18 ADHD symptoms (DSM-IV), 9 concerning inattention and 9 concerning hyperactivity/impulsivity [19]. A four-point scale is applied: 3 = several times a day, 2 = once a day, 1 = several times a week, and 0 = at the most twice a week; maximum score = 54. ADHD symptom criteria were met when at least 6 of 9 symptoms (i.e., 6/9 inattention symptoms and/or 6/9 hyperactivity/impulsivity symptoms [1]) occurred once a day or more [14,18].

**Figure 1:**
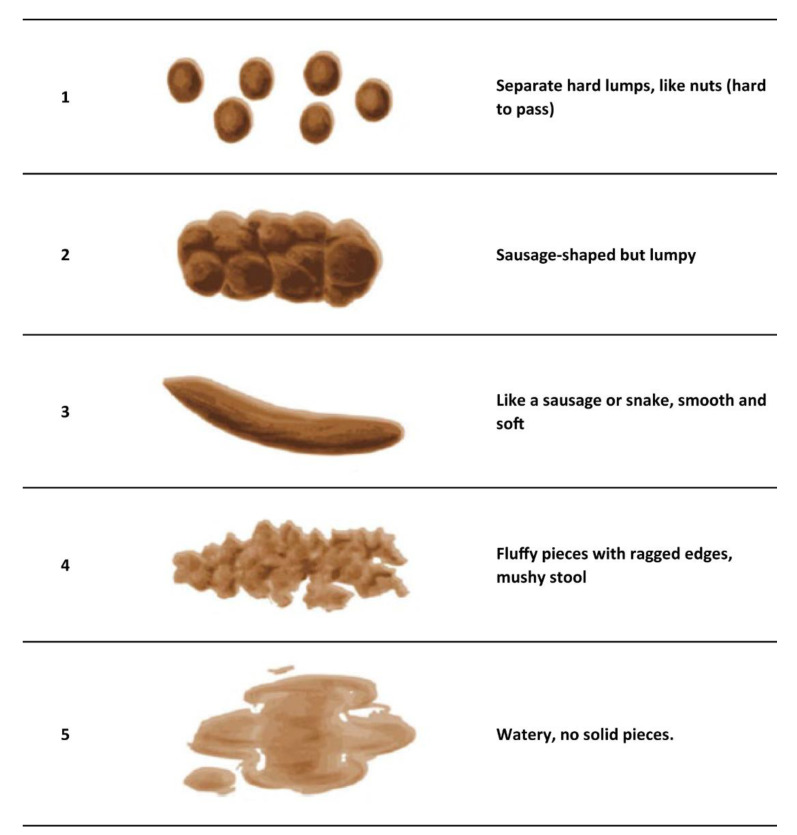
Stool types in the modified Bristol stool form scale for children (mBSFS-C [21], figure reproduced with permission).

Children participating in the BRAIN study also completed the modified Bristol stool form scale for children (mBSFS-C) in the weeks preceding T_start_ and T_end_. This scale consists of five categorical stool consistency types, varying from ‘separate hard lumps (hard to pass)’ (type 1) to ‘watery’ (type 5), and is valid and reliable for use by children [21] (Figure 1). Children had to tick off which type of stool occurred in the morning, afternoon, and/or evening. If a single passing was indicated to have the characteristics of two stool types (e.g., type 2 and 3), then the intermediate half (i.e., 2.5) was used in our analyses. For further analyses, the 5 consistency types were divided into three categories: hard stool (type 1.0–1.5–2.0), normal stool (type 2.5–3.0–3.5), and watery stool (type 4.0–4.5–5.0). Stool frequency included all bowel movements in a week. When none of the types were ticked off for an entire day, that day was counted as a “day without stool”.

### 2.4. Statistical Analysis

For the primary objective, exact logistic regression was applied, with the presence/absence of each physical complaint after the intervention (at T_end_) as the outcome variable and intervention (FFD or control) as the dependent variable. The presence/absence of a complaint at T_start_ was added as a covariate. Results were expressed as odds ratios (ORs) with their 95% confidence interval (CI) and as effect size (ES, Cohen’s d) based on the log odds and the standard deviation of the logistic distribution [22]. Here, it is assumed that the exact ORs are valid estimators of the approximate ORs, which have a fixed variance of $\frac{\pi^{2}}{\sqrt{3}}$. A value of Cohen’s d >|0.2| is considered a small effect, >|0.5| a medium effect, and >|0.8| a large effect [23]. In this study, Cohen’s d >|0.5| was regarded as clinically relevant.

For the second objective, comparable analyses were performed. The independent variables consisted of the study (INCA or BRAIN) and the change in ARS score, i.e., an ARS score decrease of at least 40% (responder) or less than 40% (non-responder) at T_end_ compared to T_start._ Complaint presence at T_start_ was added as a covariate. An additional analysis was performed to assess the association between physical complaint presence at T_end_ and the atopic constitution of the participant, adjusted for complaint presence at T_start_.

Differences in stool consistency and frequency between T_start_ and T_end_, as well as the association with the ARS change, were evaluated using non-parametric methods (the Wilcoxon signed rank, the Kruskal–Wallis test, and the Spearman rank correlation).

SAS 9.4 was used for all analyses. *p*-values < 0.05 were considered statistically significant; an a priori correction for multiple correction was not applied as it concerned individual hypotheses [24].

## 3. Results

**Figure 2:**
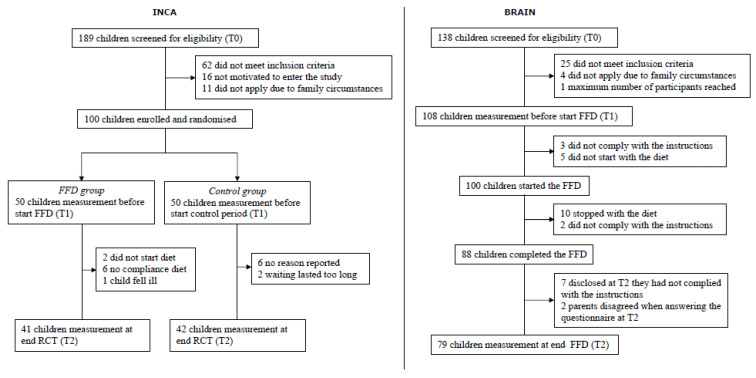
Flow charts for the INCA study and BRAIN study. INCA = Impact of Nutrition on Children with ADHD (randomised controlled trial); BRAIN = Biomarker Research in ADHD: the Impact of Nutrition (open-label trial); FFD = few-foods diet.

A total of 83 (41/42 (FFD/Control)) of the 100 INCA participants and 79 (all FFD) of the 100 BRAIN participants complied with the study protocol and completed the T_end_ measurement (Figure 2). In sum, 162 children were included in the analyses, and their baseline characteristics are shown in Table 2. Most children met the criteria for ADHD combined type (87% INCA/89% BRAIN, Fisher exact *p* = 0.93). The mean numbers of ADHD symptoms (range: 0–18) were 15.34 (INCA) and 15.96 (BRAIN) (Cohen’s d = 0.30; Kruskal–Wallis *p* = 0.0201). Regarding the total number of physical complaints at T_start_, no significant differences were established between the INCA study and the BRAIN study (Kruskal–Wallis *p* = 0.35).

### 3.1. Physical Complaints at T_end_ versus T_start_ in the INCA RCT

The INCA data were used to compare the effect of both interventions (either the FFD [FFD group] or healthy food advice [control group]) on parent-reported physical complaints. At T_start_, in 87.8% (36/41) of the children in the FFD group and 97.6% (41/42) of the children in the control group, one or more physical complaints were reported that occurred at least several times per week (Fisher exact *p* = 0.11). The presence of the 21 individual physical complaints at T_start_ and the average total number of physical complaints were not different between the FFD and control groups (Table 3). ‘Often warm’ was the most reported complaint at T_start_ and occurred in 56.1% (23/41) of the FFD group and 45.2% (19/42) of the control group. Six complaints were reported in less than 5% of the children (i.e., asthma, blotches in the face, red edged mouth, red ears, constipation, and nausea/vomiting).

A total of 10 of 21 complaints showed clinically relevant reductions in the FFD group when compared to the control group, with medium to large effect sizes (Cohen’s d) of −0.60 to −1.78. Those complaints were unusual thirst, unusual perspiration (at night or daytime), often warm, eczema, persisting cold (rhinitis), bags under eyes, diarrhoea, flatulence, problems sleeping on, and nocturnal enuresis. Seven of ten complaints had *p*-values of 0.0009–0.0357: unusual thirst, unusual perspiration (at night or daytime), often warm, persisting cold (rhinitis), diarrhoea, flatulence, and nocturnal enuresis (Table 3). For these seven complaints, Cohen’s d varied from −1.01 to −1.78. At T_end_, the total number of physical complaints was significantly decreased in the FFD group when compared to the control group (incidence rate ratio 0.50; *p* < 0.0001; Table 3).

### 3.2. Physical Complaints at T_end_ versus T_start_ in the Open-Label BRAIN Study and the INCA FFD Group

The BRAIN study (*n* = 79) results showed that 5 of 21 complaints (i.e., unusual perspiration (at night or daytime), often warm, flatulence, problems sleeping in, and problems sleeping on) decreased significantly between T_start_ and T_end_ (Table 4, Wilcoxon signed rank *p* ≤ 0.0001–0.0476). In the INCA FFD group (*n* = 41), 5 of 21 complaints decreased significantly (Wilcoxon signed rank *p* ≤ 0.0001–0.0313) between T_start_ and T_end_ (i.e., unusual thirst, unusual perspiration (at night or daytime), often warm, flatulence, and problems sleeping in). The participants in the BRAIN study and the INCA FFD group did not differ in the presence of the 21 complaints at T_start_ or T_end_ (Table 4).

Because no association of the study (BRAIN versus the INCA FFD group) with individual complaints at T_end_ was established, an additional analysis was performed combining both FFD groups, thus generating a larger sample size (*n* = 120). A total of 10 of 21 complaints showed a statistically significant decrease at T_end_ versus T_start_ (Wilcoxon *p* < 0.0001–0.0463), and 6 of 10 were in accordance with the statistically significant results of the INCA RCT, i.e., unusual thirst, unusual perspiration (at night or daytime), often warm, persisting cold, flatulence, and nocturnal enuresis. The other four complaints with a statistically significant decrease in the *n* = 120 analysis were abdominal pain, growing pain, problems sleeping in, and problems sleeping on. The total number of complaints decreased from 350 at T_start_ to 184 at T_end_ (Table 4). Due to positive correlations between several complaints (Table S1), *p*-values regarding the change in the total number of complaints would be misleading and thus are not provided.

### 3.3. Reduction in Physical Complaints in ARS Responders and Non-Responders after the FFD

The combined data of the INCA FFD group (*n* = 41) and the BRAIN participants (*n* = 79) were used to assess the association between changes in physical complaints and changes in ADHD symptoms (ARS score). At T_end_, 82/120 (68.3%) participants were classified as ARS responders (≥40% ARS score decrease) (INCA: 32/41 (78.0%); BRAIN: 50/79 (63.2%)). Analysis of the 21 individual complaints showed no significant association between the reduction in complaints at T_end_ compared to T_start_ and ARS responder status for any of the complaints, except for ‘red ears’, a complaint occurring in 2/120 children at T_start_ (Table S2). The total number of physical complaints present at T_end_, adjusted for the total number at T_start_, was significantly lower in ARS responders compared to non-responders (<40% ARS score decrease) (Table S2, incidence rate ratio 0.62, *p*-value 0.0120).

### 3.4. Additional Complaints and the mBSFS-C in the BRAIN Study

Two additional parent-reported physical complaints were assessed in the BRAIN study. ‘Daytime urinary incontinence’ was present in seven (8.8%) children at T_start_ and in five (6.3%) children at T_end_ (Wilcoxon signed rank *p* = 0.69); ‘faecal incontinence’ was reported in five (6.3%) children at T_start_ and zero (0%) children at T_end_ (Wilcoxon signed rank *p* = 0.06; Table 4). No significant difference in the reduction in these complaints was present between ARS responders and non-responders (Table S2).

The BRAIN study also provided information on stool type and frequency, measured with the mBSFS-C (Figure 1). Normal stool type increased at T_end_ compared to T_start_ (from 47.73 to 57.52%; Cohen’s d = 0.31; Wilcoxon signed rank *p* = 0.0252), while hard and watery stool types decreased (Wilcoxon signed rank *p* = 0.11 and 0.22, respectively; Cohen’s d = −0.24 and −0.15, respectively) (Table S3A). Stool frequency per week was lower at T_end_ (5.04 [SD 2.13]) compared to T_start_ (8.59 [SD 2.91]), Cohen’s d = −1.39; Wilcoxon signed rank *p*-value < 0.0001). Furthermore, at T_end_, more days without stool were reported compared to T_start_ (2.68 versus 0.92 days; Wilcoxon signed rank *p*-value <0.0001; Cohen’s d = 1.29). Stool type and frequency at T_end_ (adjusted for T_start_) were not significantly associated with ARS score decrease (Table S3B).

### 3.5. Physical Complaints and ARS Response in Children with Allergies or Atopic Constitution

In 22 of 120 children participating in the INCA FFD group (11/41) and the BRAIN study (11/79), an allergy test had been performed before the start of the FFD. At T_start_, no significant difference was established in the total number of physical complaints between children with a positive (*n* = 7) and a negative (*n* = 15) allergy test (Cohen’s d = 0.39; Fisher exact *p* = 0.21). A total of 3 of 7 (43%) children with a positive and 10 of 15 (67%) children with a negative allergy test were ARS responders (Fisher exact *p* = 0.38).

A total of 71 of 119 children (one missing value) had an atopic constitution (INCA FFD group 20/41; BRAIN study 51/79), while 48 children had a non-atopic background. At T_start_, atopic and non-atopic children did not significantly differ in either the presence of individual complaints or the total number of complaints (Table S4).

At T_end_, after following the FFD, no significant difference in ARS score decrease was established between atopic and non-atopic children; 44/71 (62%) atopic and 37/48 (77.1%) non-atopic children were ARS responders (Fisher exact *p* = 0.18; Cohen’s d = −0.36).

## 4. Discussion

The primary objective was to analyse the effect of an FFD on comorbid physical complaints in children with ADHD. We first analysed the data from the INCA RCT. Most of the odds ratios (i.e., 14/20) were <1.0 (Table 3), indicating that the FFD reduced the probability of a complaint being present at T_end_ compared to the control group. A total of 10 of 21 complaints showed a clinically relevant reduction in the FFD group when compared to the control group (Cohen’s d −0.59 to −1.77, i.e., medium to large effect sizes); seven of ten were statistically significant (*p*-values 0.0009–0.0357). Unusual thirst, unusual perspiration (at night or daytime), and often warm were the most predominant complaints at T_start_ (present in 20–50% of the children) and showed the largest effect sizes (Cohen’s d = −1.01 to −1.65) and lowest *p*-values (0.0009–0.0075). In addition, the total number of physical complaints at T_end_ was significantly reduced in the FFD group compared to the control group (60.7% vs. 18.9%, incidence rate ratio 0.50, *p* < 0.0001).

These results are in accordance with the previous RCT by Pelsser et al. [17], who also reported a significant decrease in physical complaints in the FFD group compared to the control group. In this 2010 RCT that included 24 children, the complaints were grouped into eight domains (i.e., pain, unusual thirst/unusual perspiration, eczema, asthma/rhinitis, skin problems, tiredness, gastrointestinal problems, and sleep complaints), thus preventing the direct comparison of individual complaints with the current results. Therefore, the 2010 RCT data were re-analysed at the complaint level (Table S5). A total of 12 of 21 complaints had a medium to large ES (Cohen’s d = −0.61 to −1.27) in the FFD group when compared to the control group. Those 12 complaints were abdominal pain, unusual thirst, unusual perspiration (at night or daytime), often warm, eczema, blotches in the face, red ears, often tired, diarrhoea, flatulence, problems sleeping in, and problems sleeping on; one complaint (unusual thirst) was statistically significant (*p* = 0.048).

When comparing the individual complaint results of FFD groups versus control groups in both RCTs, the changes in 7 of 21 physical complaints (i.e., unusual thirst, unusual perspiration (at night or daytime) often warm, eczema, diarrhoea, flatulence, and problems sleeping on) showed a medium to large ES in both the INCA RCT and the 2010 RCT. In the INCA study, reductions in five of seven complaints were statistically significant compared to one of seven in the 2010 RCT. However, the sample size in the 2010 RCT (*n* = 24) was considerably lower than in the INCA study (*n* = 83), which affects statistical power since *p*-values are highly influenced by the sample size; the ES, in contrast, is less affected [23]. This is illustrated by the differences in statistical significance between INCA and the 2010 RCT, while the ES-values are commensurable. Addressing clinical relevance in research is important for evidence-based practice, especially in studies with small sample sizes, which more often lead to statistically nonsignificant results but which can show effect sizes of clinical relevance [25,26]. Indeed, the reported effect sizes in similar RCTs provide adequate information for comparison of the study outcomes [25,26,27].

In sum, the combined clinically relevant results of both RCTs show an association between the FFD and decreases in specific comorbid physical complaints (unusual thirst, unusual perspiration (at night or daytime), often warm, eczema, diarrhoea, flatulence, and problems sleeping on) in children with ADHD (Table 5). The association between the FFD and the decrease in thermoregulation problems and gastrointestinal problems are particularly notable.

Thermoregulation problems (unusual thirst, unusual perspiration (at night or daytime), and often warm) were reported at T_start,_ by 22–56% of the children participating in the INCA study. These complaints showed a clinically relevant decrease in the INCA FFD group when compared to the control group (Cohen’s d = −1.01 to −1.65; *p* = 0.0009–0.0075). In the 2010 RCT, these complaints were reported in 38–50% of participating children and also showed clinically relevant improvements in the FFD group when compared to the control group (Cohen’s d = −0.86 to −1.17; *p* = 0.0476–0.40). Thermoregulation problems may be due to increased sympathetic activation triggered by stress [28]. It is unlikely that ADHD acts as the stressor in these children because non-responders, i.e., children still meeting the criteria for ADHD at the end of the FFD, showed decreases in thermoregulation problems comparable to those of the ARS responders. However, the stressor may be a component of the child’s original diet, eliminated when following the FFD. Also, considering the impact of microbial dysbiosis on paediatric diseases [29], the stressor may be related to gut microbiome dysbiosis, dissolved as a consequence of the change in diet.

Gastrointestinal problems (diarrhoea and flatulence) were reported at T_start,_ in 9.6–22.9% of the participating children in the INCA study. Both complaints decreased clinically relevantly in the FFD group when compared to the control group (Cohen’s d = −1.39 to −1.78; *p* = 0.015–0.0357). It must be stated that the complaint ‘diarrhoea’, present at T_start_ in 8/83 children, was reported in only 2/8 children in the FFD group and in 6/8 in the control group. At T_end,_ diarrhoea was reported in none of the FFD group children but in all control group children. Despite these striking results, the number of children in the FFD group would be too small to draw conclusions. In the 2010 RCT, diarrhoea and flatulence were present in 21–25% of children, showing medium to large ESs (Cohen’s d = −0.66 to −1.01) when comparing the FFD group to the control group. At T_start,_ ‘diarrhoea’ was present in 5/24 (21%) children: 2/5 in the FFD group and 3/5 in the control group. Comparable to the INCA results, diarrhoea was reported in none of the FFD group children but in all control group children at T_end_. The combined results of both the INCA RCT and the 2010 RCT point toward an association between a decrease in gastrointestinal problems and the FFD. However, studies including larger numbers of children with gastrointestinal problems are needed to confirm these results.

We also analysed the data from the open-label BRAIN study. Commensurate with the results of the two RCTs, the open-label BRAIN study (*n* = 79) results showed that thermoregulation-related complaints, gastrointestinal complaints, and sleep problems decreased significantly between T_start_ and T_end_ (Table 4, Wilcoxon signed rank *p* ≤ 0.0001–0.0476). The BRAIN results thus confirm the RCT results, pointing to a clinically relevant and statistically significant association between the FFD and the decrease in specific complaints reported in children with ADHD. Furthermore, of the two additional complaints that were included in the BRAIN study (daytime urinary incontinence and faecal incontinence), ‘faecal incontinence’ was reported in five (6.3%) children at T_start_ and zero (0%) children at T_end_. This notable decrease warrants further research into the effect of the FFD on faecal incontinence.

Children participating in the BRAIN study also filled in the mBSFS-C, showing that normal stool type increased (Cohen’s d = 0.31; *p* = 0.0252) and stool frequency decreased at the end of the FFD (Cohen’s d = −1.39; *p* < 0.0001). Only the changes in stool frequency were clinically relevant based on Cohen’s d. A change in stool frequency following the FFD points towards changes in the composition of the gut microbiome, as shown in previous studies [30,31].

We also investigated whether the decrease in physical complaints was linked to the decrease in ADHD symptoms after following an FFD, using data from the INCA FFD group and the BRAIN study. Results showed that the reduction in physical complaints after following the FFD did not differ between ARS responders and non-responders (Table S1), which concurs with the results of the previous RCT [17]. Research has shown that diet composition may profoundly affect the gut microbiome and our health [32,33]. This effect is undoubtedly applicable to a very stringent diet like the FFD that only allows a few foods. Considering that the FFD was associated with a decrease in physical complaints in all children, irrespective of the FFD’s effect on a child’s ADHD, we hypothesise that the mechanism underlying the decrease in physical complaints is different from the mechanism underlying the ARS score decrease.

Since dietary changes may affect the gut microbiome to a great extent [33], and the gut microbiome often plays a role when physical complaints are concerned [32,34], it is conceivable that the profound dietary change inextricably bound up with the FFD may affect both the microbiome and physical health in children with ADHD. Considering the high prevalence of substantially impairing functional somatic complaints (reported in 4.4% of a population-based sample of Danish children [35]), and particularly of irritable bowel syndrome, one of the most common functional gastrointestinal disorders (occurring in 8.8% of children [36]), it would be interesting to investigate the effect of the FFD on all children with functional (gastrointestinal) disorders, whether suffering from ADHD or not.

Research has provided evidence for an association between atopic diseases and ADHD [37], which may explain the high percentage of children participating in INCA and BRAIN with an atopic constitution (71/119 (60%)). Furthermore, it has been hypothesised that allergies or atopic diseases might eventually result in ADHD due to excessive stress and/or cytokine release caused by the atopic condition [37,38]. However, in our study, there was no significant association of ARS response with allergy test results or with the existence of an atopic constitution. These results suggest that, in ARS responders, the comorbid allergic and/or atopic conditions do not underly ADHD. In addition, the number of physical complaints at the start and end of the trials included in this study did not differ between the atopic and non-atopic groups, which is in accordance with the results of the 2010 RCT. Consequently, the presence of an allergic or atopic constitution did not predict whether or not a child would show a decrease in physical complaints or ADHD symptoms following the FFD. Since the number of allergic children included in the current study was small, further research into the effect of the FFD on allergies and atopic diseases in children with ADHD is advised.

Finally, in children with ADHD, comorbidity with somatic and/or atopic conditions is common [39]. However, assessments of physical complaints are not standardly applied in patients with ADHD [40]. Adding the PCQ to the anamnestic procedure in children with ADHD may improve health care and may lead to additional therapies, especially in children still suffering from physical complaints after following the FFD.

## 5. Strengths/Limitations

The strengths of this study are the availability of three separate studies (two RCTs and one open-label trial), the large sample sizes of INCA (*n* = 83) and BRAIN (*n* = 79), the inclusion of both boys and girls (INCA), and the wide range of the age group included in INCA and BRAIN together (4–10 years old).

This study has several limitations. First, the PCQ that measures 21 physical problems is a non-validated questionnaire. This questionnaire was chosen because it includes many different comorbid physical complaints and has been previously used in diet research [17] in ADHD patients, thus enabling outcome comparisons. Other questionnaires used in ADHD research include only a few heterogeneous medical conditions, e.g., the Pittsburgh side effect rating scale, which is aimed at medication side effects [41], and the NICHQ Vanderbilt ADHD assessment scale (see https://www.nichq.org/sites/default/files/resource-file/NICHQ-Vanderbilt-Assessment-Scales.pdf [accessed on 20 June 2022]). In addition, using a validated questionnaire like the children’s somatisation inventory [42], which assesses functional (i.e., medically unexplained) conditions in children, would result in missing sleep, allergy, and thermoregulation problems.

Second, like most questionnaires, the PCQ focuses on symptom presence or absence, neither considering the impairment associated with the complaint nor making an inventory of previous medical diagnostic research or therapies related to the complaint. In practice, clinical relevance is not just based on Cohen’s d but depends on the context and the outcome measure [25]. In our study, a value of Cohen’s d > |0.5| was defined as clinically relevant. Furthermore, it is conceivable that the impact of the physical complaints concerned may be different per complaint and per individual. For example, gastrointestinal complaints and eczema may be more distressing than excessive sweating and thirst. Therefore, in future research, the individual impediment resulting from each complaint and previous diagnostic research should ideally be taken into account as well.

Third, in both INCA and BRAIN, the average ARS scores at T_start_ (46.55 [INCA] and 46.19 [BRAIN]) were above the 98th percentile (i.e., 40.0 [boys, age 5–7] and 42.4 [boys, age 8–10]) [19], indicating major ADHD problems in the participating children. This severity of ADHD may not reflect the probably more moderate ADHD level in the general population of children with ADHD.

Fourth, both INCA and BRAIN investigated the short-term effect of an FFD on physical complaints. Long-term studies, in which, after following the FFD, foods are added one by one to determine which foods result in the reappearance of the physical complaints involved, are needed to investigate the long-term effects and to compose individual diets that are as elaborate as possible.

In this study, *p*-values were a priori not adjusted for the testing of multiple complaints (*n* = 21); in this case, this is not considered a limitation because the hypothesis of the effect of the FFD on physical complaints is based on the individual complaints, and it is not a matter of disjunction or conjunction testing [24]. Moreover, complaints were already investigated in the 2010 RCT [17]; in the current study, we focused on confirming these results using the data from the INCA and BRAIN studies.

## 6. Conclusions

The results of this study, which included an RCT (INCA) and an open-label study (BRAIN), confirm the results of a previous smaller RCT and show a clinically relevant and statistically significant decrease in several physical complaints in children with ADHD after following an FFD. The results point towards an association between the FFD and a decrease in thermoregulation problems, gastrointestinal complaints, eczema, and sleep problems. Further research is warranted, especially into the effect of the FFD on gastrointestinal problems, both in children with and without ADHD.

## Figures and Tables

**Table 1:** Physical complaints included in the physical complaint questionnaire ^1^.

Nr ^2^	Complaint
1	Headache
2	Abdominal pain
3	Growing pain
4	Unusual thirst
5	Unusual perspiration (at night or daytime)
6	Often warm
7	Eczema
8	Asthma
9	Persisting cold (rhinitis)
10	Blotches in the face
11	Red edged mouth
12	Red ears
13	Bags under eyes
14	Often tired
15	Diarrhoea
16	Constipation
17	Flatulence
18	Nausea/vomiting
19	Problems sleeping in
20	Problems sleeping on
21	Nocturnal enuresis
22	Daytime urinary incontinence
23	Faecal incontinence

^1^ Pelsser, L.M. et al. [17]. ^2^ 1-21: INCA and BRAIN; 22–23: BRAIN only. INCA = Impact of Nutrition on Children with ADHD, a randomised controlled trial. BRAIN = Biomarker Research in ADHD: the Impact of Nutrition, an open-label trial.

**Table 2:** Baseline (T_start_) characteristics of children included in the INCA study and BRAIN study.

	INCA				BRAIN	
	FFD *n* = 41	Control *n* = 42	*p*-Value FFD vs. Control	Total FFD + Control, *n* = 83	FFD *n* = 79	*p*-Value INCA (*n* = 83) vs. BRAIN (*n* = 79)
Boys	35 (85%)	38 (90%)	0.52 ^1^	73 (88%)	79 (100%)	0.0015 ^1^
Mean age (SD)	6.83 (1.34)	7.14 (1.19)	0.28 ^2^	6.99 (1.27)	9.21 (0.85)	<0.0001 ^2^
Atopic constitution family ^3^						
Non-atopic	20 (49%)	18 (43%)	0.66 ^1^	38 (46%)	28 (35%)	0.20 ^1^
Atopic	20 (49%)	24 (57%)		44 (53%)	51 (65%)	
Unknown	1 (2%)	0 (0%)		1 (1%)	0 (0%)	
Allergy research in child ^4^						
No research	30 (73%)	29 (69%)	0.89 ^1^	59 (71%)	68 (86%)	0.054 ^1^
Research negative	7 (17%)	7 (17%)		14 (17%)	8 (10%)	
Research positive	4 (10%)	6 (14%)		10 (12%)	3 (4%)	
ADHD type and symptom severity						
ADHD combined type	34 (83%)	38 (90%)	0.43 ^1^	72 (87%)	70 (89%)	0.93 ^1^
ADHD inattentive type	2 (5%)	2 (5%)		4 (5%)	4 (5%)	
ADHD hyperactive-impulsive type	5 (12%)	2 (5%)		7 (8%)	5 (6%)	
Mean number of ADHD symptoms (SD) (minimum (0)–maximum (18))	14.68 (2.11) (10–18)	15.98 (1.77) (12–18)	**0.0049** ^2^	15.34 (2.04) (10–18)	15.96 (2.07) (9–18)	**0.0201** ^2^
Mean ARS score (SD) (minimum (0)–maximum (54))	45.22 (4.78) (35–54)	47.86 (3.67) (41–54)	**0.0167** ^2^	46.55 (4.43) (35–54)	46.19 (5.76) (28–54)	0.78 ^2^
Total number of physical complaints ^5^ (mean; minimum-maximum)	117 (2.85; 0–8)	159 (3.79; 0–9)		276 (3.33; 0–9)	233 (2.95; 0–9)	

FFD = few-foods diet; INCA = Impact of Nutrition on Children with ADHD (a randomised controlled trial); BRAIN = Biomarker Research in ADHD: the Impact of Nutrition (an open-label trial); ADHD = Attention-deficit hyperactivity disorder. ^1^ Fisher exact test. ^2^ Kruskal–Wallis test. ^3^ At least one parent or sibling with allergy complaints such as asthma, eczema, or hay fever in history. ^4^ Patch test or blood test. ^5^ Based on complaints 1–21 listed in Table 1.

**Table 3:** Distribution and statistical analysis of presence of 21 physical complaints in the few-foods diet (FFD) group and control group of the INCA study, scored using the physical complaint questionnaire ^1^.

	FFD, *n* = 41		Control, *n* = 42		FFD vs. Control	Association of Treatment (FFD vs. Control) with Complaint at T_end_		
Complaint	T_start_ *n* (%)	T_end_ *n* (%)	T_start_ *n* (%)	T_end_ *n* (%)	T_start_ *p*-Value ^2^	Odds Ratio (95% CI) ^3^	*p*-Value	Cohen’s d
Headache	6 (14.6)	4 (9.8)	8 (19.0)	3 (7.1)	0.77	1.89 (0.24; 17.52)	0.76	0.35
Abdominal pain	11 (26.8)	5 (12.2)	16 (38.1)	11 (26.2)	0.35	0.45 (0.09; 1.94)	0.36	−0.44
Growing pain	4 (9.8)	1 (2.4)	2 (4.8)	1 (2.4)	0.43	0.41 (0.003; 52.13)	1.00	−0.49
Unusual thirst	15 (36.6)	2 (4.9)	17 (40.5)	13 (31.0)	0.82	0.05 (0.004; 0.38)	**0.0009**	−1.65
Unusual perspiration (at night or daytime)	9 (22.0)	1 (2.4)	18 (42.9)	14 (33.3)	0.06	0.06 (0.001; 0.50)	**0.0036**	−1.55
Often warm	23 (56.1)	7 (17.1)	19 (45.2)	15 (35.7)	0.38	0.16 (0.03; 0.66)	**0.0075**	−1.01
Eczema	4 (9.8)	1 (2.4)	4 (9.5)	4 (9.5)	1.00	0.15 (0.00; 1.26)	0.07	−1.05
Asthma	0 (0.0)	0 (0.0)	0 (0.0)	1 (2.4)	NA ^4^	1.02 (0.00; 19.46)	0.51	0.01
Persisting cold (rhinitis)	3 (7.3)	0 (0.0)	7 (16.7)	7 (16.7)	0.31	0.04 (0.00; 0.38)	**0.0083**	−1.78
Blotches in the face	0 (0.0)	0 (0.0)	0 (0.0)	0 (0.0)	NA	NA	NA	NA
Red edged mouth	0 (0.0)	1 (2.4)	1 (2.4)	1 (2.4)	1.00	1.00 (0.05; infinite)	0.50	0.00
Red ears	0 (0.0)	2 (4.9)	2 (4.8)	2 (4.8)	0.49	2.40 (0.28; infinite)	0.25	0.48
Bags under eyes	4 (9.8)	1 (2.4)	11 (26.2)	10 (23.8)	0.09	0.05 (<0.001; 1.15)	0.07	−1.65
Often tired	10 (24.4)	12 (29.3)	13 (31.0)	13 (31.0)	0.63	1.24 (0.31; 5.47)	0.98	0.12
Diarrhoea	2 (4.9)	0 (0.0)	6 (14.3)	6 (14.3)	0.26	0.08 (0.00; 0.79)	**0.0357**	−1.39
Constipation	0 (0.0)	1 (2.4)	1 (2.4)	1 (2.4)	1.00	1.00 (0.05; infinite)	0.50	0.00
Flatulence	7 (17.1)	1 (2.4)	7 (16.7)	7 (16.7)	1.00	0.04 (<0.001; 0.65)	**0.0151**	−1.78
Nausea/vomiting	0 (0.0)	1 (2.4)	3 (7.1)	3 (7.1)	0.24	0.95 (0.05; infinite)	0.51	−0.03
Problems sleeping in	10 (24.4)	4 (9.8)	17 40.5)	9 (21.4)	0.16	0.60 (0.09; 3.71)	0.80	−0.28
Problems sleeping on	7 (17.1)	2 (4.9)	3 (7.1)	3 (7.1)	0.19	0.34 (0.02; 4.03)	0.59	−0.60
Nocturnal enuresis	2 (4.9)	0 (0.0)	4 (9.5)	5 (11.9)	0.68	0.10 (0.00; 0.79)	**0.0329**	−1.27
Total number of physical complaints (average; minimum-maximum)	117 (2.9; 0–8)	46 (1.1; 0–6)	159 (3.8; 0–9)	129 (3.1; 0–9)	0.47	0.44 (0.31; 0.62) ^5^	**<0.0001** ^5^	NA

INCA = Impact of Nutrition on Children with ADHD. ^1^ Pelsser, L.M. et al. [17]. ^2^ Kruskal–Wallis test. ^3^ Exact logistic regression model: presence at T_end_ = presence at T_start_ + treatment group. ^4^ NA = not available. ^5^ Incidence rate ratio based on the Poisson regression model: number present at T_end_ = number present at T_start_ + study. Complaints in red font occurred in less than 5% of children, both at T_start_ and at T_end._

**Table 4:** Distribution and statistical analysis of the presence of 21 physical complaints in the INCA and BRAIN studies, scored using the physical complaint questionnaire ^1^ before (T_start_) and after (T_end_) following the few-foods diet.

	BRAIN (*n* = 79) (All Participating Children)			INCA (*n* = 41) (FFD Group Only)			Association of Study (BRAIN vs. INCA) with Complaint at T_end_		INCA + BRAIN (*n* = 120)		
Complaint	T_start_ *n* (%)	T_end_ *n* (%)	*p*-Value ^2^	T_start_ *n* (%)	T_end_ *n* (%)	*p*-Value ^2^	Odds Ratio	*p*-Value ^3^	T_start_ *n* (%)	T_end_ *n* (%)	*p*-Value ^2^
Headache	6 (7.6)	8 (10.1)	0.77	6 (14.6)	4 (9.8)	0.69	1.19 (0.29; 5.97)	1.00	12 (10.0)	12 (10.0)	1.00
Abdominal pain	20 (25.3)	15 (19.0)	0.29	11 (26.8)	5 (12.2)	0.11	1.77 (0.53; 6.96)	0.46	31 (25.8)	20 (16.7)	**0.0463**
Growing pain	6 (7.6)	1 (1.3)	0.13	4 (9.8)	1 (2.4)	0.25	0.58 (0.01; 46.05)	1.00	10 (8.3)	2 (1.7)	**0.0215**
Unusual thirst	19 (24.1)	10 (12.7)	0.06	15 (36.6)	2 (4.9)	**0.0002**	3.7 (0.69; 38.36)	0.17	34 (28.3)	12 (10.0)	**<0.0001**
Unusual perspiration (at night or daytime)	29 (36.7)	11 (13.9)	**<0.0001**	9 (22.0)	1 (2.4)	**0.0078**	5.01 (0.63; 230.91)	0.19	38 (31.7)	12 (10.0)	**<0.0001**
Often warm	41 (51.9)	15 (19.0)	**<0.0001**	23 (56.1)	7 (17.1)	**<0.0001**	1.22 (0.40; 4.05)	0.91	64 (53.3)	22 (18.3)	**<0.0001**
Eczema	1 (1.3)	0 (0.0)	1.00	4 (9.8)	1 (2.4)	0.25	4 (0.00; 76.00)	0.80	5 (4.2)	1 (0.8)	0.13
Asthma	0 (0.0)	0 (0.0)	NA	0 (0.0)	0 (0.0)	NA	NA	NA	0 (0.0)	0 (0.0)	NA
Persisting cold (rhinitis)	6 (7.6)	2 (2.5)	0.13	3 (7.3)	0 (0.0)	0.25	1.35 (0.14; infinite)	0.42	9 (7.5)	2 (1.7)	**0.0156**
Blotches in the face	1 (1.3)	3 (3.8)	0.50	0 (0.0)	0 (0.0)	NA	1.28 (0.15; infinite)	0.43	1 (0.8)	3 (2.5)	0.50
Red edged mouth	4 (5.1)	5 (6.3)	1.00	0 (0.0)	1 (2.4)	1.00	2.24 (0.21; 113.7)	0.84	4 (3.3)	6 (5.0)	0.73
Red ears	2 (2.5)	2 (2.5)	1.00	0 (0.0)	2 (4.9)	0.50	0.26 (0.004; 5.12)	0.55	2 (1.7)	4 (3.3)	0.63
Bags under eyes	10 (12.7)	11 (13.9)	1.00	4 (9.8)	1 (2.4)	0.25	6.38 (0.82; 293.48)	0.09	14 (11.7)	12 (10.0)	0.80
Often tired	16 (20.3)	26 (32.9)	0.07	10 (24.4)	12 (29.3)	0.75	1.23 (0.50; 3.14)	0.78	26 (21.7)	38 (31.7)	0.06
Diarrhoea	5 (6.3)	2 (2.5)	0.38	2 (4.9)	0 (0.0)	0.50	1.12 (0.13; infinite)	0.47	7 (5.8)	2 (1.7)	0.13
Constipation	1 (1.3)	2 (2.5)	1.00	0 (0.0)	1 (2.4)	1.00	0.52 (0.007; 41.79)	1.00	1 (0.8)	3 (2.5)	0.50
Flatulence	22 (27.8)	3 (3.8)	**<0.0001**	7 (17.1)	1 (2.4)	**0.0313**	1.14 (0.08; 64.77)	1.00	29 (24.2)	4 (3.3)	**<0.0001**
Nausea/vomiting	2 (2.5)	2 (2.5)	1.00	0 (0.0)	1 (2.4)	1.00	1.07 (0.06; 64.48)	1.00	2 (1.7)	3 (2.5)	1.00
Problems sleeping in	23 (29.1)	13 (16.5)	**0.0476**	10 (24.4)	4 (9.8)	**0.0313**	1.73 (0.47; 7.97)	0.55	33 (27.5)	17 (14.2)	**0.0030**
Problems sleeping on	11 (13.9)	4 (5.1)	**0.0391**	7 (17.1)	2 (4.9)	0.18	1.14 (0.15; 13.59)	1.00	18 (15.0)	6 (5.0)	**0.0075**
Nocturnal enuresis	8 (10.1)	3 (3.8)	0.06	2 (4.9)	0 (0.0)	0.50	1.13 (0.12; infinite)	0.47	10 (8.3)	3 (2.5)	0.0156
Total number of physical complaints (average; minimum-maximum)	233 (2.95; 0–9)	138 (1.74; 0–8)	NV	117 (2.85; 0–8)	46 (1.12; 0–6)	NV	1.60 ^4^ (1.04; 2.47)	**0.0338** ^4^	350 (2.92; 0–9)	184 (1.53; 0–8)	NV
Daytime urinary incontinence ^5^	7 (8.9)	5 (6.3)	0.69								
Faecal incontinence ^5^	5 (6.3)	0 (0.0)	0.06								

BRAIN = Biomarker Research in ADHD: the Impact of Nutrition. INCA = Impact of Nutrition on Children with ADHD. NA = not available; NV = not valid due to dependency of some complaints. ^1^ Pelsser, L.M. et al. [17]. ^2^ Wilcoxon signed rank on the differences in the presence of complaints at T_start_ and T_end._^3^ Exact logistic regression model: presence at T_end_ = presence at T_start_ + study. ^4^ Incidence rate ratio and *p*-value based on the Poisson regression model: number present at T_end_ = number present at T_start_ + study. ^5^ BRAIN study only (*n* = 79). Complaints in red font occurred in less than 5% of children, both at T_start_ and at T_end._

**Table 5:** Clinical relevance of associations between physical complaints and the few-foods diet in two RCTs, scored using the physical complaint questionnaire ^1^ before (T_start_) and after (T_end_) following the few-foods diet.

Physical Complaints	INCA RCT	2010 RCT
Headache	0	0
Abdominal pain	0	+
Growing pain	0	0
Unusual thirst	+	+
Unusual perspiration (at night or daytime)	+	+
Often warm	+	+
Eczema	+	+
Asthma	0	NA
Persisting cold (rhinitis)	+	NA
Blotches in the face	NA	+
Red edged mouth	0	0
Red ears	0	+
Bags under eyes	+	0
Often tired	0	+
Diarrhoea	+	+
Constipation	0	NA
Flatulence	+	+
Nausea/vomiting	0	NA
Problems sleeping in	0	+
Problems sleeping on	+	+
Nocturnal enuresis	+	NA
Total number of physical complaints (average; minimum-maximum)	+	+

^1^ Pelsser, L.M. et al. [17]. RCT = randomised controlled trial. INCA = Impact of Nutrition on Children with ADHD. A score of 0 = no relevant association (Cohen’s d > −0.50). + = relevant association (Cohen’s d < −0.50). NA = not available.

## Data Availability

The data presented in this study are openly available at DANS, https://doi.org/10.17026/dans-zgw-3yqc (accessed on 20 July 2022).

## Supplementary Materials

The following supporting information can be downloaded at: https://www.mdpi.com/article/10.3390/nu14153036/s1, Table S1: Spearman rank correlations (rho) between physical complaints at T_start_, grouped in domains, in the combined INCA and BRAIN study (*n* = 162); Table S2: Distribution and statistical analysis of the presence of 21 (INCA FFD group (*n* = 41)) and 23 (BRAIN study (*n* = 79)) physical complaints, scored using the physical complaint questionnaire, comparing responders (decrease on the ADHD rating scale (ARS) score ≥ 40%) and non-responders (ARS score decrease < 40%) before (T_start_) and after (T_end_) following the few-foods diet; Table S3A: Stool type and frequency measured by the modified Bristol stool form scale for children^1^ before (T_start_) and after (T_end_) following the few-foods diet, in the BRAIN study (*n* = 79); Table S3B: Association of stool type and frequency measured by the modified Bristol stool form scale for children^1^, with changes in ADHD symptoms measured by the ADHD Rating Scale (ARS) before (T_start_) and after (T_end_) following the few-foods diet, in the BRAIN study (*n* = 79); Table S4: Distribution and statistical analysis of the presence of 21 physical complaints in children with and without an atopic constitution who participated in the INCA FFD group (*n* = 41) and the BRAIN study (*n* = 79). Data for one of the INCA participants are missing; Table S5: Distribution and statistical analysis of the presence of 21 physical complaints, scored using the physical complaint questionnaire^1^, in the few-foods diet (FFD) group and the control group of the 2010 RCT^1^ (*n* = 24).
