# Supplementary material for: Physical Complaints Decrease after Following a Few-Foods Diet in Children with ADHD
Source: Nutrients. 2022 Jul 24;14(15):3036. doi: 10.3390/nu14153036 (PMC9332265; doi:10.3390/nu14153036)
Supplement: Supplementary file 1 [file nutrients-14-03036-s001.zip › nutrients-1804786-supplementary.pdf]

Table S1. Spearman rank correlations (rho) between physical complaints at T_{start}, grouped in domains, in the combined INCA and BRAIN study (n=162).

Domain	Complaints		rho	P-value
Thermoregulation	Unusual perspiration	Unusual thirst	0.21	0.0085
	Unusual perspiration	Often warm	0.16	0.0373
	Often warm	Unusual thirst	0.24	0.0025
Gastrointestinal complaints	Diarrhoea	Flatulence	0.12	0.14
	Diarrhoea	Abdominal pain	0.06	0.44
	Flatulence	Abdominal pain	0.31	0.0001
Pain	Headache	Abdominal pain	0.09	0.25
	Headache	Growing pain	0.04	0.64
	Growing pain	Abdominal pain	0.23	0.0026
Fatigue	Tired	Eyebags	0.48	0.0001
	Tired	Problems sleeping in	0.16	0.0487
	Tired	Problems sleeping on	0.17	0.0310
	Eyebags	Problems sleeping in	0.16	0.0440
	Eyebags	Problems sleeping on	0.19	0.0148
	Problems sleeping in	Problems sleeping on	0.10	0.21

Table S2. Distribution and statistical analysis of presence of 21 (INCA FFD group [n=41]) and 23 (BRAIN study [n=79]) physical complaints, scored using the Physical Complaint Questionnaire[1], comparing responders (score decrease on the ADHD rating scale (ARS) ≥ 40%) and non-responders (ARS score decrease < 40%) before (T_{start}) and after (T_{end}) following the few-foods diet.

	Responders (n=82)		Non-responders (n=38)		Association of ARS score decrease (responder vs non-responder) with complaint at T_{end}		Association of study (BRAIN vs INCA) with complaint at T_{end}	
**Physical complaint**	T_{start} n (%)	T_{end} n (%)	T_{start} n (%)	T_{end} n (%)	Odds ratio (95% CI)[2]	P-value	Odds ratio (95% CI)[2]	P-value
**Headache**	10 (12.2)	7 (8.5)	2 (5.3)	5 (13.2)	0.54 (0.13; 2.41)	0.51	1.10 (0.26; 5.58)	1.00
**Abdominal pain**	19 (23.2)	12 (14.6)	12 (31.6)	8 (21.1)	0.76 (0.25; 2.49)	0.79	1.71 (0.51; 6.74)	0.50
**Growing pain**	6 (7.3)	1 (1.2)	4 (10.5)	1 (2.6)	0.47 (0.01; 40.58)	1.00	0.49 (0.01; 42.25)	1.00
**Unusual thirst**	26 (31.7)	7 (8.5)	8 (21.1)	5 (13.2)	0.52 (0.11; 2.40)	0.49	3.59 (0.66; 37.52)	0.18
**Unusual perspiration (at night or daytime)**	25 (30.5)	6 (7.3)	13 (34.2)	6 (15.8)	0.52 (0.12; 2.35)	0.50	4.26 (0.52; 199.6)	0.28
**Often warm**	43 (52.4)	15 (18.3)	21 (55.3)	7 (18.4)	1.06 (0.35; 3.55)	1.00	1.23 (0.40; 4.09)	0.90
**Eczema**	4 (4.9)	1 (1.2)	1 (2.6)	0 (0.0)	0.33 (0.02; infinite)	0.75	3.00 (0.00; 57.00)	0.75
**Asthma**	0 (0.0)	0 (0.0)	0 (0.0)	0 (0.0)	NA[3]	NA	NA	NA
**Persisting cold (rhinitis)**	5 (6.1)	1 (1.2)	4 (10.5)	1 (2.6)	1.00 (0.01; 117.3)	1.00	1.19 (0.14; infinite)	0.45
**Blotches in the face**	1 (1.2)	1 (1.2)	0 (0.0)	2 (5.3)	0.24 (0.00; 2.03)	0.14	0.76 (0.09; infinite)	0.58
**Red edged mouth**	2 (2.4)	2 (2.4)	2 (5.3)	4 (10.5)	0.26 (0.02; 1.94)	0.24	1.81 (0.16; 93.94)	1.00
**Red ears**	2 (2.4)	1 (1.2)	0 (0.0)	3 (7.9)	0.08 (0.00; 0.58)	**0.0165**	0.14 (0.002; 2.92)	0.27
**Bags under eyes**	9 (11.0)	6 (7.3)	5 (13.2)	6 (15.8))	0.55 (0.12; 2.53)	0.55	5.38 (0.67; 250.2)	0.16
**Often tired**	21 (25.6)	23 (28.1)	5 (13.2)	15 (39.5)	0.54 (0.21; 1.35)	0.21	1.13 (0.45; 2.92)	0.94
**Diarrhoea**	6 (7.3)	1 (1.2)	1 (2.6)	1 (2.6)	0.39 (0.01; 33.67)	1.00	0.90 (0.10; infinite)	0.53
**Constipation**	1 (1.2)	2 (2.4)	0 (0.0)	1 (2.6)	0.41 (0.005; 34.24)	1.00	0.45 (0.005; 37.78)	1.00
**Flatulence**	21 (25.6)	4 (4.9)	8 (21.1)	0 (0.0)	2.25 (0.37; infinite)	0.25	1.25 (0.08; 73.63)	1.00
**Nausea/vomiting**	2 (2.4)	1 (1.2)	0 (0.0)	2 (5.3)	0.23 (0.004; 4.63)	0.49	(0.81 0.039;51.64)	1.00
**Problems sleeping in**	24 (29.3)	11 (13.4)	9 (23.7)	6 (15.8)	0.81 (0.23; 3.01)	0.91	1.67 (0.45; 7.74)	0.60
**Problems sleeping on**	12 (14.6)	4 (4.9)	6 (15.8)	2 (5.3)	0.97 (0.13; 11.23)	1.00	1.14 (0.15; 13.36)	1.00
**Nocturnal enuresis**	6 (7.3)	2 (2.4)	4 (10.5)	1 (2.6)	1.29 (0.04; 117.5)	1.00	1.00 (0.12; infinite)	0.50
**Total number of complaints (average; min-max)**	245 (2.99; 0-9)	108 (1.32; 0-6)	105 (2.76; 0-7)	76 (2.00; 0-8)	0.62 (0.43; 0.90)[4]	**0.0120**	1.43 (0.94; 2.17)[4]	0.09
Daytime urinary incontinence[5]	3 (6.0)	2 (4.0)	4 (13.8)	3 (10.3)	0.54 (0.04; 7.72)	0.90		
Faecal incontinence[5]	1 (2.0)	0 (0.0)	4 (13.8)	0 (0.0)	NA	NA		

[1] Pelsser, L.M., et al., Effects of food on physical and sleep complaints in children with ADHD: a randomised controlled pilot study. Eur J Pediatr, 2010. 169(9): p. 1129-38

[2] Exact logistic regression model: Presence at T_{end} = Presence at T_{start} + ARS responder status + study

[3] NA=not available (maximum likelihood estimate does not exist)

[4] Incidence rate ratio based on Poison regression model: Number present at T_{end} = Number present at T_{start} + ARS responder status + Study

[5] BRAIN study only (50 responders, 29 non-responders)

ARS=ADHD Rating Scale

INCA=Impact of Nutrition on Children with ADHD (a randomised controlled trial); BRAIN=Biomarker Research in ADHD; the Impact of Nutrition (an open trial)

Complaints in red font occurred in less than 5% of children (n=120), both at T_{start} and at T_{end}

Table S3A. Stool type and frequency measured by the modified Bristol stool form scale for children[1] before (T_{start}) and after (T_{end}) following the few-foods diet, in the BRAIN study (n=79).

Stool type and frequency	T_{start} Mean (SD)	T_{end} Mean (SD)	T_{end} - T_{start} Mean difference (SD)	P-value[2]	Cohen's d
**Percentage normal type**	47.73 (26.12)	57.52 (36.45)	9.78 (37.22)	0.0252	0.31
**Percentage hard type**	39.87 (27.01)	32.53 (33.60)	-7.34 (36.03)	0.11	-0.24
**Percentage watery type**	12.40 (16.06)	9.95 (16.94)	-2.45 (17.54)	0.22	-0.15
**Stool count in the observation weeks**	8.59 (2.91)	5.04 (2.13)	-3.56 (3.06)	<0.0001	-1.39
**Days without stool**	0.92 (1.11)	2.68 (1.57)	1.76 (1.58)	<0.0001	1.29

[1] Lane, M.M., et al., Reliability and validity of a modified Bristol Stool Form Scale for children. J Pediatr, 2011. 159(3): p. 437-441.e1
[2] Wilcoxon signed rank test
BRAIN= Biomarker Research in ADHD; the Impact of Nutrition (an open label trial)

Table S3B. Association of stool type and frequency measured by the modified Bristol stool form scale for children[1], with change in ADHD symptoms measured by the ADHD Rating Scale (ARS), before (T_{start}) and after (T_{end}) following the few-foods diet, in the BRAIN study (n=79).

Stool type and frequency	ARS score decrease				
	**≥ 40% (responder) (n=50)**	**< 40% (non-responder) (n=29)**	**Difference**	**P-value[2]**	**Cohen's d**
**Percentage normal type**	Mean (SD)	Mean (SD)			
T_{start}	48.61 (25.86)	46.20 (26.95)	2.41	0.79	0.09
T_{end}	54.12 (39.51)	63.37 (30.52)	-9.26	-	-
Difference	5.50 (39.22)	17.17 (32.84)		0.17	-0.32
P-value[3]	0.31	0.0075			
**Percentage hard type**	Mean (SD)	Mean (SD)			
T_{start}	38.62 (25.33)	42.02 (30.04)	-3.39	0.85	-0.12
T_{end}	33.99 (35.67)	30.01 (30.13)	3.98	-	-
Difference	-4.63 (37.56)	-12.01 (33.35)		0.49	-0.47
P-value[3]	0.44	0.09			
**Percentage watery type**	Mean (SD)	Mean (SD)			
T_{start}	12.76 (15.46)	11.78 (17.30)	0.98	0.66	0.06
T_{end}	11.88 (19.31)	6.61 (11.36)	5.27	-	-
Difference	-0.87 (17.62)	-5.17 (17.38)		0.41	0.25
P-value[3]	0.71	0.12			
**Stool count in the observation weeks**	Mean (SD)	Mean (SD)			
T_{start}	8.60 (3.05)	8.59 (2.71)	0.01	0.86	0.00
T_{end}	5.10 (2.06)	4.93 (2.27)	0.17	-	-
Difference	-3.50 (3.18)	-3.66 (2.88)	0.16	0.63	0.05
P-value[3]	<0.0001	<0.0001			

[1] Lane, M.M., et al., Reliability and validity of a modified Bristol Stool Form Scale for children. J Pediatr, 2011. 159(3): p. 437-441.e1
[2] Kruskal-Wallis test
[3] Wilcoxon signed rank test
BRAIN= Biomarker Research in ADHD; the Impact of Nutrition (an open label trial)

Table S4. Distribution and statistical analysis of presence of 21 physical complaints in children with and without an atopic constitution, participating in the INCA FFD group (n=41) and the BRAIN study (n=79). Data of one INCA participant is missing.

Physical complaint	Atopic constitution (n=71) T_{start} n (%)	Atopic constitution (n=71) T_{end} n (%)	Non-atopic constitution (n=48) T_{start} n (%)	Non-atopic constitution (n=48) T_{end} n (%)	Tstart P-value[1]	Association of group (atopic vs non-atopic) with complaint at T_{end}: Odds ratio[2]	P-value	Cohen's d
**Headache**	6 (8.5)	8 (11.3)	6 (12.5)	4 (8.3)	0.54	1.51	0.75	0.23
**Abdominal pain**	21 (29.6)	15 (21.1	10 (20.8)	5 (10.4)	0.39	2.08	0.29	0.40
**Growing pain**	5 (7.04)	2 (2.8)	5 (10.4)	0 (0.00)	0.52	1.98	0.30	0.38
**Unusual thirst**	22 (40.0)	9 (12.7)	12 (25.0)	3 (6.6)	0.54	2.01	0.49	0.39
**Unusual perspiration (at night or daytime)**	20 (28.3)	5 (7.0)	18 (37.5)	7 (14.6)	0.32	0.52	0.47	-0.36
**Often warm**	39 (54.9)	12 (16.9)	24 (50.0)	9 (18.8)	0.71	0.81	0.87	-0.12
**Eczema**	5 (7.0)	0 (0.0)	0 (0.0)	1 (1.4)	0.08	NA	NA	NA
**Asthma**	0 (0.0)	0 (0.0)	0 (0.0)	0 (0.0)	NA[3]	NA	NA	NA
**Persisting cold (rhinitis)**	4 (5.6)	0 (0.0)	5 (10.4)	2 (4.2)	0.48	0.44	0.28	-0.45
**Blotches in the face**	0 (0.0)	2 (2.8)	1 (2.1)	1 (2.1)	0.40	1.61	0.36	0.26
**Red edged mouth**	4 (5.6)	5 (7.0)	0 (0.0)	1 (2.1)	0.15	2.96	0.61	0.60
**Red ears**	1 (1.4)	1 (1.4)	3 (4.2)	1 (2.1)	1.00	2.26	0.86	0.45
**Bags under eyes**	8 (11.4)	8 (11.4)	6 (12.5)	4 (8.3)	1.00	1.49	0.79	0.22
**Often tired**	16 (22.5)	27 (38.0)	9 (18.8)	11 (22.9)	0.65	2.01	0.14	0.39
**Diarrhoea**	3 (4.2)	0 (0.0)	4 (8.3)	2 (4.2)	1.00	0.29	0.17	-0.68
**Constipation**	1 (1.4)	2 (2.8)	0 (0.0)	1 (2.1)	1.00	0.68	1.00	-0.21
**Flatulence**	1 (1.4)	3 (4.2)	0 (0.0)	0 (0.0)	1.00	2.04	0.96	0.39
**Nausea/vomiting**	2 (2.8)	2 (2.8)	0 (0.0)	1 (2.1)	0.51	1.40	1.00	0.19
**Problems sleeping in**	19 (26.8)	12 (16.9)	13 (27.1)	5 (10.4)	1.00	1.80	0.46	0.32
**Problems sleeping on**	14 (19.7)	5 (7.0)	4 (8.3)	1 (1.4)	0.12	2.65	0.69	0.54
**Nocturnal enuresis**	7 (9.9)	2 (2.8)	2 (4.2)	1 (2.1)	0.31	0.45	1.00	-0.44
**Total number of physical complaints (average; minimum-maximum)**	215 (3.0; 0-8)	123 (1.7; 0-8)	130 (2.7; 0-9)	60 (1.3; 0-7)	0.29	1.33[4]	0.16[4]	NA

[1] Fisher exact test
[2] Exact logistic regression model: Presence at Tend = Presence at Tstart + atopic group
[3] NA=not available
[4] Incidence rate ratio and P-value based on Poisson regression model: Number present at T_{end} = Number present at T_{start}+ treatment group
Complaints in red font occurred in less than 5% of children (n=120), both at T_{start} and at T_{end}

Table S5. Distribution and statistical analysis of presence of 21 physical complaints, scored using the Physical Complaint Questionnaire[1], in the few-foods diet (FFD) group and control group of the 2010 RCT[1] (n=24).

	FFD (n=13)		Control (n=11)		FFD vs Control	Association of treatment (FFD vs Control) with complaint at T_{end}		
**Physical complaint**	**T_{start} n (%)**	**T_{end} n (%)**	**T_{start} n (%)**	**T_{end} n (%)**	**T_{start} P-value[2]**	**Odds Ratio (95% CI)[3]**	**P-value**	**Cohen's d**
**Headache**	2 (15.4)	0 (0.0)	5 (45.5)	3 (27.3)	0.18	0.45 (0.00; 4.48)	0.29	-0.44
**Abdominal pain**	8 (61.5)	1 (7.7)	8 (72.7)	5 (45.5)	0.68	0.10 (0.00; 1.47)	0.12	-1.27
**Growing pain**	0 (0.0)	0 (0.0)	0 (0.0)	1 (9.1)	NA	0.85 (0.00; 16.1)	0.46	-0.09
**Unusual thirst**	5 (38.5)	0 (0.0)	4 (36.4)	3 (27.3)	1.00	0.12 (0.00; 0.97)	**0.0476**	-1.17
**Unusual perspiration (at night or daytime)**	8 (61.5)	0 (0.0)	4 (36.4)	2 (18.2)	0.41	0.16 (0.00; 1.53)	0.09	-1.01
**Often warm**	5 (38.5)	3 (23.1)	6 (54.5)	6 (54.5)	0.68	0.21 (0.00; 3.23)	0.40	-0.86
**Eczema**	1 (7.7)	0 (0.0)	5 (45.5)	5 (45.5)	0.06	0.20 (0.00; 3.80)	0.17	-0.89
**Asthma**	1 (7.7)	1 (7.7)	1 (9.1)	1 (9.1)	1.00	NA[4]	NA	NA
**Persisting cold (rhinitis)**	1 (7.7)	0 (0.0)	1 (9.1)	0 (0.0)	1.00	NA	NA	NA
**Blotches in the face**	1 (7.7)	0 (0.0)	3 (27.3)	3 (27.3)	0.30	0.33 (0.00-6.33)	0.25	-0.61
**Red edged mouth**	1 (7.7)	0 (0.0)	2 (18.2)	2 (18.2)	0.58	0.50 (0.00-9.50)	0.33	-0.38
**Red ears**	2 (15.4)	0 (0.0)	3 (27.3)	3 (27.3)	0.63	0.16 (0.00-1.71)	0.10	-1.01
**Bags under eyes**	8 (61.5)	4 (36.4)[5]	3 (27.3)	3 (27.3)	0.12	0.48 (0.00; 4.05)	0.29	-0.41
**Often tired**	9 (69.2)	4 (33.3)[6]	3 (27.3)	4 (36.4)	0.10	0.24 (0.00; 1.63)	0.12	-0.79
**Diarrhoea**	2 (15.4)	0 (0.0)	3 (27.3)	3 (27.3)	0.63	0.16 (0.00; 1.71)	0.10	-1.01
**Constipation**	1 (7.7)	1 (7.7)	0 (0.0)	0 (0.0)	1.00	NA	NA	NA
**Flatulence**	3 (23.1)	1 (7.7)	3 (27.3)	3 (27.3)	1.00	0.30 (0.00; 3.11)	0.20	-0.66
**Nausea/vomiting**	1 (7.7)	0 (0.0)	0 (0.0)	0 (0.0)	1.00	NA	NA	NA
**Problems sleeping in**	4 (30.8)	1 (7.7)	4 (36.4)	4 (36.4)	1.00	0.15 (0.00; 1.26)	0.07	-1.05
**Problems sleeping on**	2 (15.4)	0 (0.0)	2 (18.2)	2 (18.2)	1.00	0.24 (0.00; 2.80)	0.17	- 0.79
**Nocturnal enuresis**	3 (23.1)	0 (0.0)	0 (0.0)	0 (0.0)	0.22	NA	NA	NA
**Total number of physical complaints (average; min-max)**	68 (5.23; 2-10)	16 (1.23; 0-4)	60 (4.64; 0-13)	53 (4.82; 0-12)	0.27	0.33[7] (0.17; 0.66)	0.0014[7]	NA

[1] Pelsser, L.M., et al., Effects of food on physical and sleep complaints in children with ADHD: a randomised controlled pilot study. Eur J Pediatr, 2010. 169(9): p. 1129-38
[2] Fisher exact test
[3] Exact logistic regression model: Presence at T_{end} = Presence at T_{start} + treatment group
[4] NA=not available (maximum likelihood estimate does not exist)
[5] n=11 due to 2 missing values
[6] n=12 due to 1 missing value
[7] Incidence rate ratio and P-value based on Poisson regression model: Number present at T_{end} = Number present at T_{start} + treatment group
RCT=randomised controlled trial
